# Identification of Ftr1 and Zrt1 as iron and zinc micronutrient transceptors for activation of the PKA pathway in *Saccharomyces cerevisiae*

**DOI:** 10.15698/mic2017.03.561

**Published:** 2017-03-02

**Authors:** Joep Schothorst, Griet V. Zeebroeck, Johan M. Thevelein

**Affiliations:** 1 Laboratory of Molecular Cell Biology, Institute of Botany and Microbiology, KU Leuven, Belgium.; 2 Department of Molecular Microbiology, VIB, Kasteelpark Arenberg 31, B-3001 Leuven-Heverlee, Flanders, Belgium.

**Keywords:** Ftr1, Zrt1, iron, zinc, transceptor, signaling, PKA

## Abstract

Multiple types of nutrient transceptors, membrane proteins that combine a transporter and receptor function, have now been established in a variety of organisms. However, so far all established transceptors utilize one of the macronutrients, glucose, amino acids, ammonium, nitrate, phosphate or sulfate, as substrate. This is also true for the *Saccharomyces cerevisiae* transceptors mediating activation of the PKA pathway upon re-addition of a macronutrient to glucose-repressed cells starved for that nutrient, re-establishing a fermentable growth medium. We now show that the yeast high-affinity iron transporter Ftr1 and high-affinity zinc transporter Zrt1 function as transceptors for the micronutrients iron and zinc*. *We show that replenishment of iron to iron-starved cells or zinc to zinc-starved cells triggers within 1-2 minutes a rapid surge in trehalase activity, a well-established PKA target. The activation with iron is dependent on Ftr1 and with zinc on Zrt1, and we show that it is independent of intracellular iron and zinc levels. Similar to the transceptors for macronutrients, Ftr1 and Zrt1 are strongly induced upon iron and zinc starvation, respectively, and they are rapidly downregulated by substrate-induced endocytosis. Our results suggest that transceptor-mediated signaling to the PKA pathway may occur in all cases where glucose-repressed yeast cells have been starved first for an essential nutrient, causing arrest of growth and low activity of the PKA pathway, and subsequently replenished with the lacking nutrient to re-establish a fermentable growth medium. The broadness of the phenomenon also makes it likely that nutrient transceptors use a common mechanism for signaling to the PKA pathway.

## INTRODUCTION

As a unicellular eukaryotic micro-organism, the yeast *Saccharomyces cerevisiae *has to be able to rapidly adapt to severe fluctuations in the extracellular conditions. One of the main fluctuating parameters in the extracellular environment is nutrient availability. Hence, microorganisms like yeast have developed a multitude of complex nutrient-sensing mechanisms in order to properly respond to fluctuations in the supply of specific nutrients and to adapt cellular metabolism, growth and development accordingly. Among eukaryotic microorganisms, these mechanisms have by far been elucidated in greatest detail in *S. cerevisiae* 1,2. One of the most common adaptation mechanisms with respect to nutrient availability is the induction of high-affinity transporters for specific essential nutrients lacking or limiting in the medium, so that trace amounts of the sparse nutrient can be taken up efficiently by the microorganism. Examples of this adaptation mechanism have been described in yeast for all essential nutrients, i.e. the macronutrients glucose, nitrogen, phosphate and sulfate, and micronutrients like metal ions and vitamins. Limitation for glucose causes upregulation of Hxt6 and Hxt7 3, for nitrogen the general amino acid permease Gap1 4 and ammonium transporters Mep1 and Mep 2 5, for phosphate Pho84 6, for sulfate Sul1 and Sul2 7, for iron Ftr1 8 and for Zinc Zrt1 9. Even for non-essential nutrients like uracil, uracil permease is induced in media lacking uracil 10. The high-affinity transporters generally undergo rapid downregulation upon re-exposure to their substrate through endocytic internalization, which is induced by ubiquitination, followed by sorting to the multivesicular body (MVB) and subsequently by vacuolar degradation 10,11,12,13,14.

Next to the upregulation of high-affinity transporters in response to starvation for specific nutrients, the yeast *S. cerevisiae *has a multitude of different nutrient-sensing pathways at its disposal 1,2. One of these nutrient-sensing pathways is the Fermentable Growth Medium (FGM) induced pathway, which maintains high activity of PKA in a medium with a fermentable sugar and all other nutrients required for growth. Deprivation for any of the other essential nutrients in the presence of a fermentable sugar causes growth arrest and downregulation of PKA activity, while subsequent re-addition of the missing nutrient triggers rapid modulation of PKA targets, such as activation of the enzyme trehalase by phosphorylation, during the resumption of fermentable growth 15,16. This nutrient activation of the PKA pathway is not triggered by an increase in cAMP as in the case of glucose activation of the PKA pathway in glucose-derepressed yeast cells 16. The signaling mechanism remains elusive.

We have shown that activation of the PKA pathway targets by the other nutrients besides glucose, is mediated by high-affinity transporters induced during the starvation period that function as transporter-receptors or 'transceptors': Gap1 for amino acids 17,18, Pho84 for phosphate 19,20,21, Mep2 for ammonium 5 and Sul1,2 for sulfate 22. Major arguments include the activation by non-transported and/or non-metabolized analogs and the separation of transport and signaling by specific mutations. For each of these transceptors, starvation for their substrate triggers strong upregulation at the transcriptional level, while re-addition of the missing substrate triggers rapid endocytosis and vacuolar degradation. As these proteins combine a transporter function with a receptor function, we have named them transceptors 23. We have proposed that a specific conformation has to be induced in the transceptor to initiate signaling, which appears to be dependent on the structure of the substrate, since not all transported substrates are able to trigger signaling 18,24. Transceptors may constitute an intermediate step in the development of receptors from transporters during evolution 25.

After the initial identification of nutrient transceptors in *S. cerevisiae, *the concept of transceptors has spread to other organisms, ranging from yeasts and fungi to higher eukaryotic organisms including plants, mice and humans 26. However, while an increasing number of transceptors in multiple organisms has now been identified, their substrates, glucose, phosphate, amino acids, ammonium, nitrate and sulfate, all belong to the class of macronutrients. Up to now, no transceptors for micronutrients, like metal ions or vitamins, have been identified. Since starvation for an essential micronutrient also causes growth arrest and entry into stationary phase, it is expected that the PKA pathway will also be downregulated and that re-addition of the lacking micronutrient may cause a similar sudden activation of the PKA pathway as is observed after starvation and re-addition of a macronutrient. This is especially true since high-affinity transporters for micronutrients, like metal ions, are well known to be strongly induced also by starvation for their substrate and rapidly downregulated by endocytosis and breakdown in the vacuole after re-addition of the lacking micronutrient 8,9,14,27. Based on these criteria we selected two known cation transporters, the high-affinity iron transporter Ftr1 and the high-affinity zinc transporter Zrt1, as potential micronutrient transceptors.

Ftr1 is a high-affinity Fe^3+^ transporter, which together with the Fet3 oxidoreductase forms the high-affinity iron uptake system. Uptake of Fe^2+^ by Ftr1 is preceded by oxidation of Fe^2+^ to Fe^3+^, followed by inter-complex channelling of Fe^3+^ to Ftr1 and subsequent transport over the plasma membrane 8,28,29,30. The strong interplay between Ftr1 and Fet3 is also shown by the fact that they need each other for proper targeting to the plasma membrane 8. As mentioned above, *FTR1 *expression is strongly upregulated under iron limitation 8, which is controlled mainly by the two transcription factors, Aft1 and Aft2 31,32. Upon iron limitation, Aft1 accumulates in the nucleus and induces the expression of *FTR1 *and *FET3* 33. Ftr1 is also subjected to regulation at the post-translational level. When iron is again available, Ftr1 is rapidly endocytosed and degraded presumably to prevent overaccumulation of iron to toxic levels 14. Ftr1 endocytosis is mediated by ubiquitination and requires an active Ftr1-Fet3 complex. Hence, Ftr1 shows a very similar expression pattern as the macronutrient transceptors, being present at the plasma membrane under substrate limiting conditions and being rapidly removed from the plasma membrane when the substrate becomes plentiful again.

Upon zinc limitation, *S. cerevisiae *stimulates zinc uptake to ensure zinc homeostasis. *S. cerevisiae *has three different zinc uptake systems: a high-affinity zinc transporter (K_m_ ≈ 1 μM) Zrt1 9, a low-affinity zinc transporter (K_m_ ≈ 10 μM) Zrt2 34 and the non-specific Fet4 transporter 35. Expression of both *ZRT1 *and *ZRT2 *is regulated through the activity of the Zap1 transcription factor 36. The expression of *ZAP1 *is strongly stimulated in response to zinc limitation and its enhanced activity strongly induces expression of *ZRT1 *while inhibiting expression of *ZRT2 *31,37,38. In analogy with Ftr1 and the known macronutrient transceptors, the high-affinity zinc transporter Zrt1 is also rapidly removed from the plasma membrane upon exposure to high extracellular zinc concentrations 9. This process is mediated by endocytosis following ubiquitination and subsequent vacuolar degradation and can be induced by high levels of cadmium and cobalt as well 27,39.

We now show that both iron and zinc limitation lead to downregulation of PKA pathway targets during growth arrest and that re-addition of iron or zinc, respectively, triggers the same rapid activation of the PKA target trehalase as previously observed with the macronutrients. We provide evidence that signaling is independent of the intracellular iron or zinc levels, and that the high-affinity transporters, Ftr1 and Zrt1, have an additional sensing function for activation of the PKA pathway, next to their transport function. Hence, we have identified Ftr1 and Zrt1 as the first transceptors for micronutrients, which significantly broadens the concept of nutrient transceptors.

## RESULTS

### Influence of iron and zinc deprivation on PKA pathway targets and expression of Ftr1 and Zrt1

**Figure 1:**
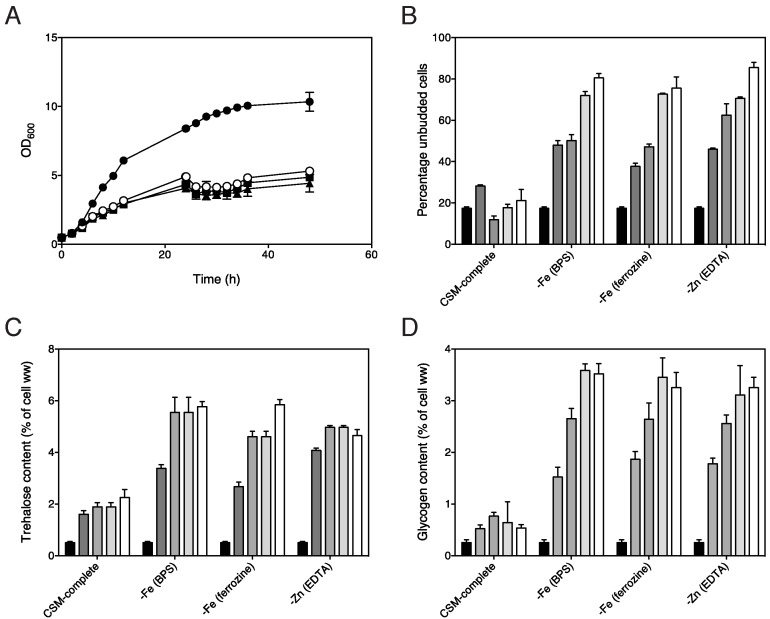
FIGURE 1: Growth rate, budding percentage, and trehalose and glycogen accumulation upon deprivation for iron or zinc. All experiments were performed with the Σ1278 wild type strain. All samples originate from a preculture that was grown overnight in all trace medium, and subsequently diluted at t = 0 to an OD_600_ of 0.5 in the indicated deprivation media. **(A)** Growth inhibition upon exposure to different iron or zinc deprivation conditions, n = 2. Cells were put on fresh deprivation medium each 24 h and OD_600_ was measured at regular intervals. (closed circles) all trace medium, (open circles) iron deprivation with BPS, (closed squares) iron deprivation with ferrozine, (closed triangles) zinc deprivation with EDTA. **(B)** Percentage of unbudded cells in response to the indicated deprivation conditions with CSM complete as control condition, n = 2. **(C)** Accumulation of trehalose in response to the indicated deprivation conditions, n = 2. **(D)** Accumulation of glycogen in response to the indicated deprivation conditions, n = 2. **(B,C,D)** The shaded bars indicate non-starved cells and 1, 2, 3 or 4 days of metal ion starvation.

We have tested the effect of different iron and zinc deprivation conditions on arrest of cell proliferation and a set of well-established read-outs for activity of the PKA pathway, in particular the accumulation of the storage carbohydrates trehalose and glycogen. Cell proliferation was rapidly inhibited upon imposing starvation for iron using an iron deprivation medium supplemented with iron specific chelators in order to neutralize minute amounts of iron present in the milliQ water used to prepare the medium (Fig. 1A). We have used the membrane impermeable chelator bathophenantroline disulfonate (BPS) and the membrane permeable chelator ferrozine/3-(2-Pyridyl)-5,6-diphenyl-1,2,4-triazine-p,p´-disulfonic acid monosodium salt hydrate) for iron deprivation. For zinc starvation we have used a medium deprived of zinc and supplemented with the non-specific ethylenediaminetetraacetic acid (EDTA) chelator. In all conditions tested, growth was virtually inhibited within 24 to 30 h after the onset of metal ion deprivation (Fig. 1A). Consistently, the percentage of unbudded cells had significantly increased after 24 h of metal ion starvation and reached about 80% after 72 h of starvation (Fig. 1B). The trehalose content rapidly increased under all metal ion deprivation conditions to reach a maximum approximately after 48 h (Fig. 1C) while glycogen content increased more slowly to reach a maximum after about 72 h (Fig. 1D). This indicates that entrance of stationary phase caused by iron or zinc deprivation is also associated with development of a low-PKA phenotype.

**Figure 2:**
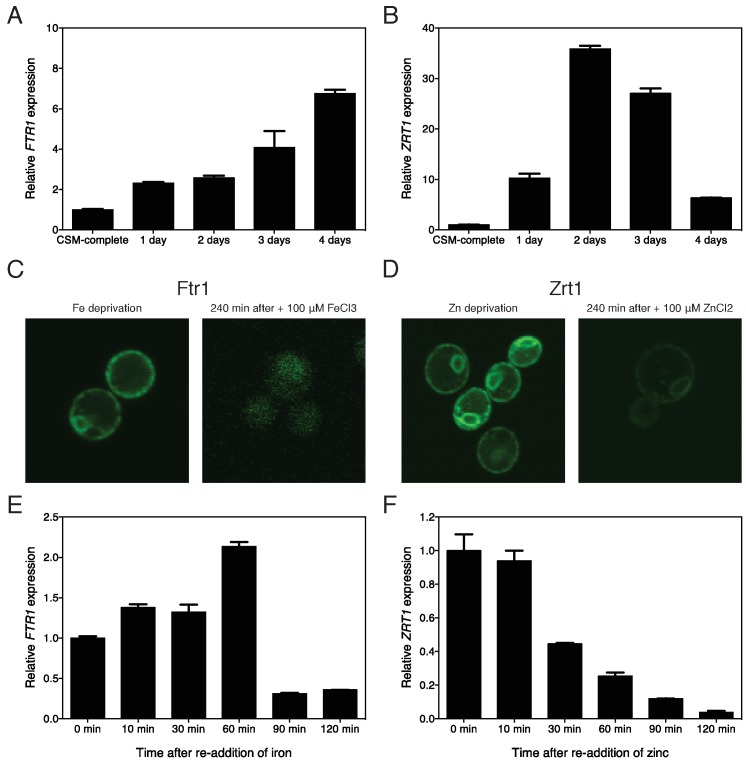
FIGURE 2: Expression level of *FTR1* and *ZRT1* and cellular localization of Ftr1 and Zrt1 upon iron or zinc deprivation and re-addition. Relative expression of *FTR1* **(A) **and *ZRT1* **(B) **in response to deprivation of iron or zinc, respectively, n = 2. As listed in Materials & Methods, a specific set of reference genes was used depending on the experiment. Localization of C-terminally tagged Ftr1-GFP **(C)** and Zrt1-GFP **(D)** under their substrate deprivation conditions (left panel) as well as after re-addition of 100 μM of the missing metal ion for 240 min (right panel), n = 2. Relative expression of *FTR1* **(E) **and *ZRT1* **(F) **at different time points after re-addition of 100 μM of the missing metal ion, iron or zinc, respectively, n = 2.

Using qPCR on RNA samples extracted from the cells, we show that under the appropriate metal ion deprivation conditions the expression of *FTR1* and *ZRT1* is strongly induced, with *FTR1* expression continuing to increase during the 4-day iron starvation period (Fig. 2A), while *ZRT1* expression reached a maximum already after 48 h of zinc starvation (Fig. 2B). *FTR1* expression was not upregulated under zinc deprivation conditions, while *ZRT1* expression was not upregulated under iron deprivation conditions supporting the specificity of the starvation conditions. We also determined the cellular localization of Ftr1 and Zrt1 using genomically GFP-tagged constructs. This showed that under iron or zinc deprivation conditions Ftr1 or Zrt1, respectively, accumulated at the plasma membrane and to a smaller extent at an intracellular membrane, possibly the ER membrane since the GFP tag might delay ER exit (Fig. 2C, D). Addition of 100 μM iron or zinc for 240 min caused virtual disappearance at the plasma membrane of the Ftr1 or Zrt1 signal, respectively (Fig. 2C, D). Transcriptional expression of *FTR1* and *ZRT1* was also downregulated after re-addition of 100 μM iron or zinc, respectively (Fig. 2E, F).

### The phenotype caused by iron or zinc deficiency is specific since it is reversed by re-addition of only iron or zinc

**Figure 3:**
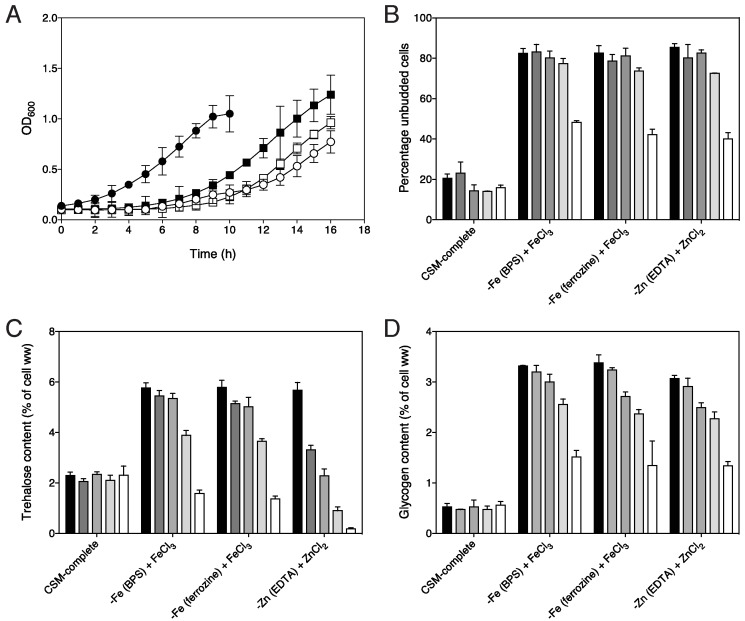
FIGURE 3: Reversal of the low-PKA phenotype developed in response to iron or zinc deprivation is specific for iron or zinc, respectively. **(A)** Growth induction by re-addition of 100 μM of either FeCl_3_ or ZnCl_2_ at t = 0 to cells that had been deprived for 4 days in the respective metal ion deprivation media. Growth was measured by determination of OD_600_. (closed circles) all trace medium, (closed squares) ferrozine iron starvation medium + FeCl_3_, (open squares) EDTA zinc starvation medium + ZnCl_2_, (open circles) BPS iron starvation medium + FeCl_3_, n = 2 **(B) **Percentage of unbudded cells after re-addition of 100 μM of either FeCl_3_ or ZnCl_2_ at t = 0 to cells that had been deprived for 4 days in the respective metal ion deprivation media indicated. The shaded bars represent 0, 2, 4, 8 and 12 h after metal ion re-addition, n = 2. **(C)** Trehalose mobilization after re-addition of 100 μM of either FeCl_3_ or ZnCl_2_ at t = 0 to cells that had been deprived for 4 days in the respective metal ion deprivation media indicated or had not been starved for iron (CSM-complete). **(D)** Glycogen mobilization after re-addition of 100 μM of either FeCl_3_ or ZnCl_2_ at t = 0 to cells that had been deprived for 4 days in the respective metal ion deprivation media indicated or had not been starved for iron (CSM-complete). **(C,D)** The shaded bars represent 0, 30, 60, 120 and 240 min after metal ion re-addition, n = 2.

We have checked the specificity of the iron and zinc starvation regime by testing the reversibility of the effect on growth, budding percentage and targets of the PKA pathway. This was especially important for zinc since we added the unspecific chelator EDTA to the zinc deprivation medium to ensure thorough zinc starvation. Re-addition of only iron or only zinc to cells starved for four days for either iron or zinc, respectively, caused in each case rapid recovery of growth (Fig. 3A), indicating that the cells were specifically arrested because of lack of either iron or zinc. Iron-induced growth recovery after BPS-elicited iron deprivation was slightly slower than after ferrozine-elicited starvation, suggesting that BPS may cause more stringent iron deprivation or may reduce to some extent the level of one or more other metal ions. The resumption of growth was correlated with a proportionate reduction in the number of unbudded cells (Fig. 3B). The storage carbohydrates trehalose and glycogen were mobilized within the first four h after re-addition of the metal ion with the drop in trehalose content being more pronounced than that in glycogen (Fig. 3C, D). Mobilization of trehalose and glycogen preceded significant recovery of growth. In contrast, addition of the same concentration of iron or zinc to non-starved cells did not result in any mobilization of trehalose or glycogen (Fig. 3C, D: CSM-complete). These results show that both growth resumption and downregulation of PKA pathway targets can be induced by re-addition of only iron to iron-starved cells and only zinc to zinc-starved cells, supporting the notion that the cells were truly starved for iron and zinc, respectively. Furthermore, for our purposes of studying metal ion induced activation of the PKA pathway, a two-day starvation period appeared to be appropriate. Iron deprivation with the BPS chelator appeared to be more reproducible than with the ferrozine chelator and was thus used in the following experiments.

### Re-addition of iron to iron-deprived cells triggers a rapid, transient Ftr1-dependent activation of trehalase

**Figure 4:**
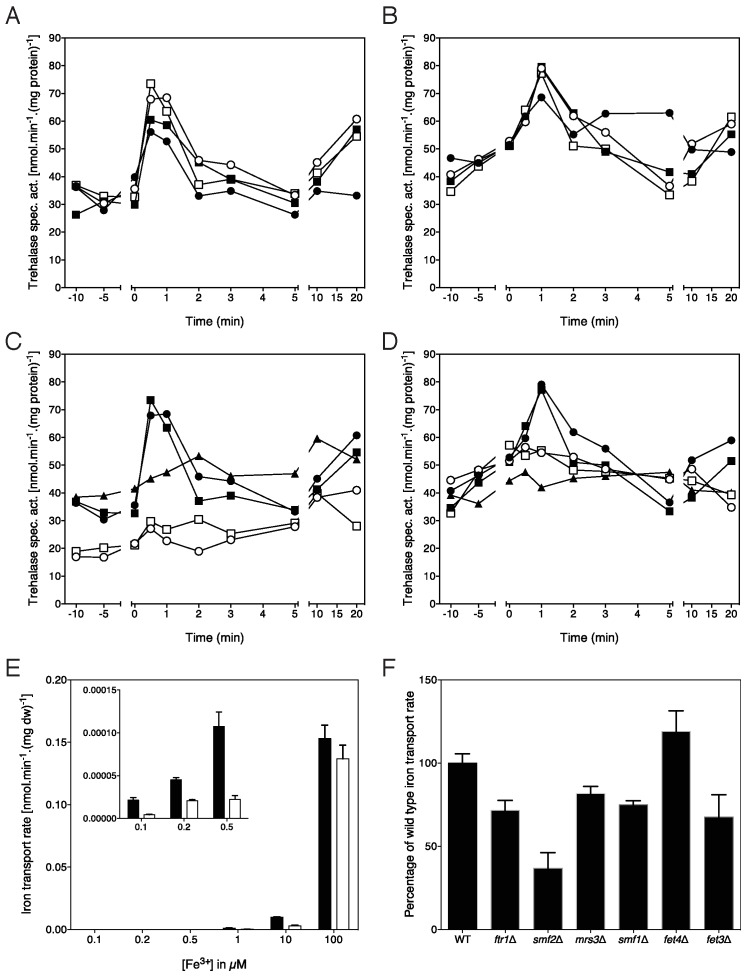
FIGURE 4: Re-addition of iron to iron-deprived cells triggers Ftr1-dependent activation of the PKA target trehalase. **(A)** Activation of trehalase in the wild type strain after addition of different concentrations of FeCl_3_ to cells deprived for iron for two days in the presence of the BPS chelator. 100 nM (closed circles), 1 μM (closed squares), 10 μM (open circles) and 100 μM (open squares) FeCl_3_. **(B) **Activation of trehalase in the wild type strain after addition of different concentrations of FeCl_2_ to cells deprived for iron for two days in the presence of the BPS chelator. 100 nM (closed circles), 1 μM (closed squares), 10 μM (open circles) and 100 μM (open squares) FeCl_2_. **(C)** Activation of trehalase in wild type, *ftr1*Δ and *fet3*Δ strains after addition of different concentrations of FeCl_3_ to cells deprived for iron for two days in the presence of the BPS chelator. 100 μM and 1 μM. Wild type, 1 μM (closed circles) and 100 μM (closed squares) FeCl_3_; *ftr1*Δ, 1 μM (open circles) and 100 μM (open squares) FeCl_3_ and *fet3*Δ, 100μM FeCl_3 _(closed triangles). **(D)** Activation of trehalase in wild type, *ftr1*Δ and *fet3*Δ strains after addition of different concentrations of FeCl_2_ to cells deprived for iron for two days in the presence of the BPS chelator. Wild type, 1 μM (closed circles) and 100 μM (closed squares) FeCl_2_; *ftr1*Δ, 1 μM (open circles) and 100 μM (open squares) FeCl_2_ and *fet3*Δ, 100μM FeCl_2 _(closed triangles). **(A-D)** All experiments were performed 3-5 times with consistent results; representative results are shown. **(E) **Uptake of different concentrations of Fe^55^Cl_3_ in the wild type (black bars) and *ftr1*Δ (white bars) strains, n = 2. The inset shows the uptake at the lower concentrations in enlarged format. **(F)** Uptake of 100 μM Fe^55^Cl_3 _in strains with a deletion of a single metal ion transporter gene, expressed as % of the uptake in the wild type strain, n = 2.

After establishing that iron and zinc deprivation resulted in a low-PKA phenotype as well as the induction and localization of Ftr1 and Zrt1 at the plasma membrane, we tested the influence of iron and zinc re-addition on short-term activation of the PKA pathway. We used the well-established downstream target trehalase as a direct read-out for PKA activity 15,22. This showed that very low iron concentrations, as low as 100 nM, in the form of FeCl_3_ or FeCl_2_ were able to trigger a rapid, transient increase in trehalase activity in cells starved for two days for iron using the BPS chelator (Fig. 4A, B). Concentrations higher than 1 μM FeCl_3_ or FeCl_2 _did not enhance trehalase activation further. Subsequently, we investigated whether this trehalase activation was dependent on the iron transporter, Ftr1. Deletion of *FTR1*, or deletion of *FET3*, which results in mislocalization of Ftr1 8, completely abolished iron-induced trehalase activation both with 1 and 100 μM FeCl_3_ (Fig. 4C) or FeCl_2 _(Fig. 4D), indicating that iron-induced signaling to the PKA pathway is dependent on Ftr1.

### Iron-induced signaling does not depend on intracellular iron entry

Next, we investigated whether Ftr1-dependent iron-induced signaling originates from the plasma membrane, or requires iron uptake in the cytoplasm triggering an intracellular iron response. The first case would suggest that Ftr1 acts as an iron transceptor while the second case would indicate an unknown intracellular iron sensing mechanism. To distinguish between the two possibilities, we measured Fe^55^ uptake upon re-addition of different iron concentrations to iron-starved cells and compared the uptake in the wild type with that in the* ftr1*Δ strain. At the high concentration of 100 μM, iron uptake in the *ftr1*Δ strain was hardly impaired compared to that in the wild type strain, while at concentrations of 10 μM and lower iron uptake was strongly reduced in the* ftr1*Δ strain (Fig. 4E). High concentrations of iron are apparently taken up also by other carriers. Since iron-induced trehalase activation was completely abolished in the *ftr1*Δ-strain at a concentration of 100 μM, we can conclude that iron entry into the cytosol is not sufficient to trigger trehalase activation and that therefore the signaling must originate from the Ftr1 carrier in the plasma membrane which apparently plays an essential role as transceptor. We also tested which other transporters could be involved in the uptake of high concentrations of iron using a series of carrier deletion strains. The *smf2*Δ strain showed the largest reduction in uptake of 100 μM iron suggesting that Smf2 is the main alternative transporter for iron uptake under these conditions (Fig. 4F).

### Re-addition of zinc to zinc-starved cells triggers a rapid, transient Zrt1-dependent activation of trehalase

**Figure 5:**
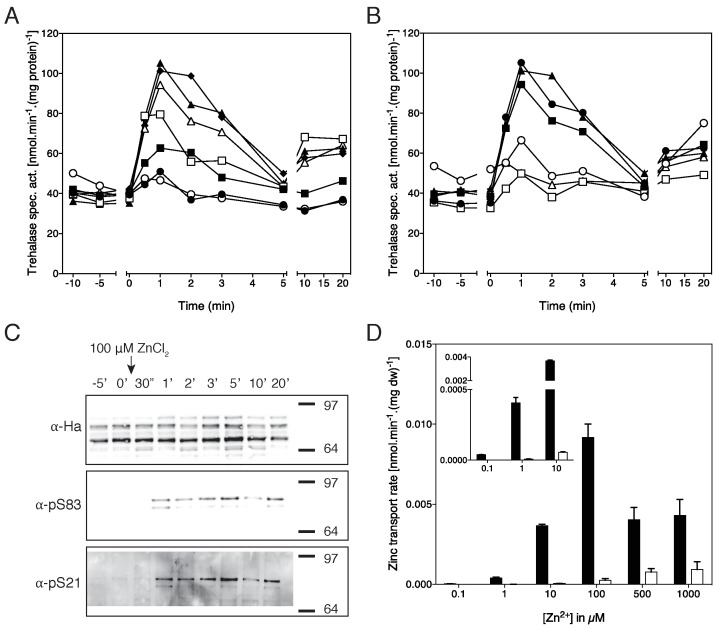
FIGURE 5: Re-addition of zinc to zinc-deprived cells triggers Zrt1-dependent activation of the PKA target trehalase. **(A)** Activation of trehalase in the wild-type strain after addition of different concentrations of ZnCl_2_ to cells deprived for zinc for two days in the presence of the EDTA chelator. 100 nM (open circles), 500 nM (closed squares), 1 μM (open squares), 10 μM (closed triangles), 100 μM (open triangles), 1 mM (closed diamonds) ZnCl_2_ and starvation medium as negative control (closed circles). **(B) **Activation of trehalase in wild-type (closed symbols) and *zrt1*Δ (open symbols) strains after addition of different concentrations of ZnCl_2_ to cells deprived for zinc for two days in the presence of the EDTA chelator. 1 μM (circles), 100 μM (squares) and 1 mM (triangles). **(A,B)** All experiments were performed 3-5 times, representative results are shown. **(C)** Phosphorylation of trehalase on the amino acid residues S21 and S83 as detected with phosphospecific antibodies in response to addition of 100 μM ZnCl_2_ to cells of the wild type strain deprived for zinc for two days in the presence of the EDTA chelator. The trehalase-HA construct was expressed from the pYX212-NTH1-HA (LEU2) plasmid in the BY4742-strain. **(D)** Uptake of different concentrations of Zn^65^Cl_2_ in the wild type (black bars) and *zrt1*Δ strains (white bars), n = 2. The inset shows the uptake at the lower concentrations in enlarged format.

Re-addition of zinc to zinc-deprived cells triggered a very similar rapid and transient increase in trehalase activity as in the case of iron re-addition to iron-starved cells (Fig. 5A). The activation was concentration-dependent, with 100 nM of ZnCl_2_ barely able to induce an increase in trehalase activity, 500 nM ZnCl_2_ caused an intermediate level of activation, while concentrations above 1 μM ZnCl_2 _triggered maximal trehalase activation. Next, we tested the importance of Zrt1 for zinc-induced trehalase activation. Deletion of *ZRT1 *resulted in a severe reduction of zinc-induced trehalase activation. Even with the higher concentrations, 10, 100 and 1000 μM ZnCl_2_, which fully activate trehalase in the wild-type strain, trehalase activation was almost absent in the *zrt1*Δ strain (Fig. 5B). As opposed to the complete absence of iron-induced trehalase activation in the *ftr1*Δ strain, some very limited residual zinc-induced trehalase activation was present in the *zrt1*Δ strain. These results indicate that zinc-induced signaling to the PKA pathway is largely dependent on the Zrt1 transporter, with some unknown transporter(s) being able to sustain slight residual signaling. We also demonstrate that zinc-induced trehalase activation is correlated with phosphorylation of trehalase on Ser21 and Ser83 using phospho-specific antibodies (Fig. 5C), as previously shown for glucose, nitrogen- and sulfate-induced trehalase activation in appropriately-starved cells 15,22. The transient increase in trehalase activity does not precisely correlate with the extent of phosphorylation. This can be explained by the fact that phosphorylation is not the only determinant of trehalase enzymatic activity 15,22. Phosphorylation is also required for binding of the 14-3-3 proteins, which strongly enhance trehalase enzymatic activity, and the Dcs1 inhibitor prevents binding of the 14-3-3 proteins (Bmh1/2) to trehalase. Hence, phosphorylation can still be high while the activity may already be reduced.

### Zinc-induced signaling does not depend on intracellular zinc entry

Next, we investigated whether zinc-induced activation of trehalase originates from the Zrt1 transporter in the plasma membrane, similar to the involvement of Ftr1 in iron-induced signaling. Hence, we compared uptake of Zn^65^Cl_2_ at different concentrations of zinc in the wild type and *zrt1*Δ strain. At all concentrations uptake of zinc in the *zrt1*Δ strain was strongly reduced and it was virtually absent at the lower concentrations (Fig. 5D). This indicates that Zrt1 is the dominant zinc transporter under our zinc deprivation conditions. However, while at low zinc concentrations, uptake was almost absent in the *zrt1*Δ strain, there was still a significant level of zinc uptake present at the higher zinc concentrations of 100 μM, 500 μM and 1 mM ZnCl_2_. Moreover, for 500 μM and 1 mM ZnCl_2 _this uptake activity was actually higher than the zinc uptake activity of the wild type strain with 1 μM ZnCl_2_ (Fig. 5D). While addition of 1 μM ZnCl_2_ in the wild type strain triggered a strong increase in trehalase activity, addition of 100 μM or 1 mM in the *zrt1*Δ strain only triggered very small residual increases in trehalase activity (Fig. 5B). Hence, as in the case of iron-induced signaling, we can conclude that entry of zinc into the cytosol is not enough to trigger signaling to the PKA pathway and that therefore the signal likely originates from the Zrt1 carrier in the plasma membrane.

### Mutagenesis of specific aspartate residues affects Zrt1 function, but was unable to fully uncouple transport and signaling capacity

**Figure 6:**
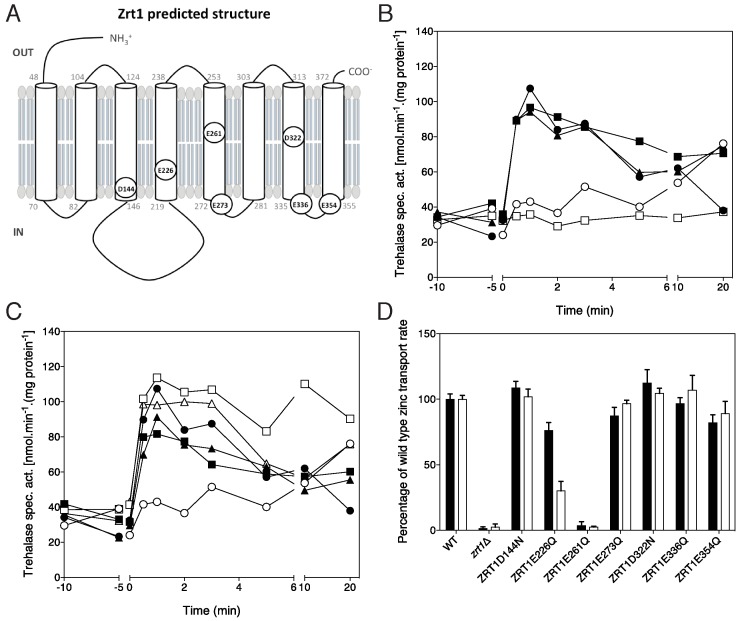
FIGURE 6: Signaling capacity and zinc uptake activity in a *zrt1*Δ strain expressing different Zrt1 alleles containing a mutated aspartate or glutamate residue. **(A) **Schematic overview of Zrt1 with the location in or close to TMDs indicated of the aspartate and glutamate residues that were mutagenized to asparagine or glutamine, respectively. **(B, C)** Activity of trehalase as a function of time after addition of 100 μM ZnCl_2_ to cells of a *zrt1*Δ strain harboring the wild type *ZRT1* gene on plasmid YCplac33-Zrt1, the empty plasmid YCplac33 or different point mutant alleles of *ZRT1* on plasmid YCplac33. All experiments were performed 3-5 times; representative results are shown. **(B) **Wild type *ZRT1* (closed circles), *zrt1*Δ (open circles), *ZRT1*^D144N^ (closed squares), *ZRT1*^E226Q^ (closed triangles) and *ZRT1*^E261Q^ (open squares). **(C) **Wild type *ZRT1* (closed circles), *zrt1*Δ (open circles), *ZRT1*^E273Q^ (closed squares), *ZRT1*^D322N^ (open squares), *ZRT1*^E336Q^ (open triangles) and *ZRT1*^E354Q^ (closed triangles). **(D) **Uptake of either 10 μM (black bars) or 100 μM (white bars) Zn^65^Cl_2_ in the same strains as used in **B, C**, n = 2.

In an attempt to obtain further evidence that Zrt1 acts as a transceptor for zinc-induced signaling to the PKA pathway, we have performed site-directed mutagenesis with the aim of specifically affecting either transport or signaling. We have focussed specifically on negatively charged amino acid residues in or near transmembrane domains since they may be involved in the binding and translocation of the positive zinc ions through the membrane. We have changed the aspartate (D) and glutamate (E) residues indicated in Fig. 6A into uncharged asparagine (N) and glutamine (Q) residues, respectively. Signaling capacity and zinc uptake activity were measured in a z*rt1*Δ strain expressing the mutant alleles from a plasmid or harboring the empty vector. In most cases the strains expressing a mutant Zrt1 allele displayed a similar extent of zinc-induced activation of trehalase compared to the strain expressing the wild type allele, except for the E261Q allele, which was completely deficient in supporting trehalase activation (Fig. 6B, C). The latter strain was also completely deficient in Zn^65^ uptake, similar to the *zrt1*Δ strain (Fig. 6D). This identifies the E261 glutamate residue, located in the middle of TMD5, as a crucial residue for Zrt1 functionality, both for transport and signaling. Interestingly, the strain expressing the E226Q allele displayed a partial reduction in zinc uptake activity with about 70% at a concentration of 100 μM ZnCl_2_ (Fig. 6D), while it showed the same extent of trehalase activation as the strain expressing the wild type allele (Fig. 6B). Hence, with the E226Q allele we observed a partial uncoupling of transport and signaling activity of Zrt1.

## DISCUSSION 

We have introduced the concept of transceptors for transporters with an additional receptor function that is linked to the transport function, in the sense that transport of the substrate through the transceptor elicits a conformational change that triggers a signaling event to the inside of the cell 23. This contrasts with signaling events that are initiated after the entry of the transporter substrate into the cell and in which the transporter only plays a role by making the substrate available for sensing by an intracellular sensor system. In recent years, more and more examples of transceptors or putative transceptors in other organisms have been reported. In all cases it concerned nutrient transceptors. The *CDT-1*, *CDT-2* and *CLP1 *genes have recently been shown to encode three putative cellodextrin transceptors in the mold *Neurospora crassa* 40,41 and the Hxt1 monosaccharide transporter been shown to have an additional sensor function in the smut *Ustilago maydis *42. Plant transceptors include a set of nitrate transceptors encoded by *LATD/NIP* 43,44, *Mt*NRT1,3 45,46, *At*NRT1.1/CHL1 47,48,49,50 and *At*NRT1.2 47,51, as well as putative ammonium transceptors such as *Lj*AMT3 52, *At*AMT1,3 53 and *At*AMT1.1 54, and a putative sulfate transceptor *At*SULTR1;2 55,56. Transceptors have been identified in other organisms, such as GT1 in *Leishmania mexicana* 57 and GLUT2 in mice 58,59,60. Finally, the concept of transceptors has also emerged in human cells with the identification of *h*CNT1 61 and *h*SNAT2 62,63,64. Hence, the concept of transceptors seems to be conserved throughout evolution in eukaryotic organisms.

A major difference between the transceptor system that we discovered in yeast for nutrient-induced activation of the PKA signaling pathway and the other transceptor systems reported in the literature, is that in our system multiple transceptors for different nutrients signal to the same target pathway, the PKA pathway. This is a classical signal transduction pathway that is not directly related to the transport or the metabolism of the nutrient concerned, but rather controls a variety of physiological and developmental properties of yeast cells. Our current discovery of Ftr1 and Zrt1 acting as transceptors for the micronutrients iron and zinc, respectively, makes it very likely that all these transceptors use the same signaling mechanism for activation of the PKA pathway. This should facilitate its elucidation. Another common characteristic of the transceptors acting on the PKA pathway is that the high sensitivity for signaling by the nutrient matches with the high affinity for transport of the nutrient, suggesting that the goal of the signaling is related to the function of the ligand as a nutrient. Apparently, the transceptor signals to the cellular machinery that a deficient essential nutrient has become available again and that the fermentable growth medium is thus again complete. Since this provides the most optimal growth medium for the species *S. cerevisiae,* fermentation and cell proliferation should be stimulated and storage sugar and stress tolerance mechanisms downregulated. The cellular machinery and physiology should be focussed on generating offspring as rapidly as possible.

Iron- and zinc-induced activation of trehalase was weaker and also more transient compared to activation with the macronutrients nitrogen, phosphate and sulfate. This may have to do with the extent to which the cells can enter stationary phase and therefore acquire a low-PKA status upon iron or zinc deprivation. Since all the other nutrients are still present, and since all major nutrients are well-established ligands for activation of the PKA pathway, starvation for only iron or only zinc in some way has to be able to shut off activation of PKA by all the other nutrients. Starvation for a macronutrient may be more powerful in this respect. We also know from extensive previous work on optimizing the starvation conditions required for observation of subsequent maximal nutrient-induced activation of trehalase that deep entrance into G0 with a clear low PKA phenotype is essential for a pronounced subsequent response. This may explain why it was more difficult to establish the proper starvation conditions for iron and zinc than with the macronutrients nitrogen, phosphate and sulfate. Another possible explanation is the high toxicity of metal ions and thus the need to curtail metal ion influx into the cytosol to avoid accumulation of excessive levels of metal ions. A similar rapid and transient activation of trehalase was observed after addition of ammonium to nitrogen-starved cells 5, which may be related in a similar way to the possible detrimental effect of ammonium on the intracellular pH.

The high toxicity of metal ions was a possible concern for the interpretation of our results. However, the data in Fig. 3A show that addition of 100 μM of either FeCl_3_ or ZnCl_2_ to appropriately starved cells caused resumption of growth, contradicting a strong toxic effect under the conditions in which we measured signaling and transport. Also, short-term, relatively simple responses like post-translational trehalase activation may be less sensitive to high levels of metal ions than more complex, long-term responses like gene expression or physiological read-outs.

Because the response to re-addition of the micronutrients iron and zinc was less pronounced than with the macronutrients nitrogen, phosphate and sulfate, we have concentrated on trehalase activation as a read-out for activation of the PKA pathway, rather than longer-term responses, like changes in gene expression and in physiological properties, that have previously been used for that purpose. Nutrient-induced trehalase activation is both the most sensitive, the most rapid and most specific target of PKA activity known in yeast. The mechanism involved has been elucidated in great detail and it is very well established that the increase in trehalase activity is dependent on phosphorylation by PKA 15,65,66,67.

The main goal of the present paper was to elucidate whether the high-affinity transporters Ftr1 for iron and Zrt1 for zinc, acted as transceptors for iron- and zinc-induced activation of the PKA pathway in appropriately starved cells, as previously demonstrated for the high-affinity transporters Gap1 17, Mep2 5, Pho84 19 and Sul1,2 22 for amino acid-, ammonium-, phosphate- and sulfate-induced activation in appropriately starved cells. To achieve this goal, different experimental approaches were tried in order to uncouple signaling from transport, in the sense that signaling still happens without entry of the substrate into the cytosol or that the substrate enters into the cytosol through another way, without signaling being triggered. The finding that deletion of Ftr1 or Zrt1 eliminated iron- or zinc-induced signaling, respectively, argues against involvement of a specific receptor for the metal ions, as was previously found for glucose activation of the cAMP-PKA pathway by the glucose- and sucrose-sensing G-protein coupled receptor Gpr1 68. The next challenge was to discriminate between the requirement of Ftr1 and Zrt1 as transporter-receptors, initiating the signaling upon transport of the metal ion through the protein transmembrane passageway, or merely as regular transporters that have to bring the metal ion into the cell so that it can be sensed by an intracellular sensing mechanism.

The finding that for high iron concentrations, the uptake in the *ftr1*Δ strain was not significantly different from that in the wild type strain, indicated that Ftr1 itself rather than intracellular iron acted as trigger for the signaling. This is consistent with all previous work on the nutrient transceptors involved in activation of the PKA pathway in yeast that the transceptor itself acts as a nutrient receptor and that the nutrient does not have to enter the cytosol to trigger signaling 1. Our results confirm earlier observations that in addition to Ftr1 other non-specific iron uptake mechanisms are also upregulated under iron deprivation conditions 31. This explains why Ftr1 is not essential for uptake of high levels of iron under our conditions. The Fet3 dependency of signaling is likely due to the requirement of Fet3 for proper localization of Ftr1 at the plasma membrane. In principle, we cannot exclude the possibility that Fet3 itself would act as the iron sensor. However, this seems rather unlikely since it functions as an oxidoreductase and the other transceptors for activation of the PKA pathway in yeast, including Zrt1, do not require such an enzyme for functionality. A role of Ftr1 as iron sensor, on the other hand, fits very well with the thoroughly established transceptor concept for activation of the PKA pathway by the other nutrients.

We found that re-addition of both Fe^2+^ and Fe^3+^ to iron-starved cells could trigger trehalase activation with equal efficiency. This was surprising as it was suggested in the current model for high-affinity uptake that Fe^2+^ is oxidized by Fet3 to Fe^3+^ and subsequently channelled inside the protein complex to Ftr1 for uptake 30. Hence, in this model, Fe^2+^ itself does not act as a substrate for Ftr1. Our finding might imply that Fe^3+^ could directly act as a signaling substrate of Ftr1, although we cannot exclude that it is first reduced to Fe^2+^ by iron reductases like Fre1 present at the plasma membrane, before being oxidized back to Fe^3+^ by Fet3 and channelled to Ftr1 for uptake and signaling. Hence, for uptake Fe^2+^ may have to be oxidized to Fe^3+^, while for signaling it might not be required. Although previous work showed that for the Gap1 and Pho84 transceptors, the same substrate binding site is used for transport and signaling, other data using non-transported substrate analogues showed that complete transport is not required for signaling 18,20,69.

To establish Zrt1 as a zinc transceptor, we could use a similar argumentation as in the case of Ftr1. Although in this case, deletion of Zrt1 reduced zinc uptake at all concentrations, there was a level of uptake left in the *zrt1*Δ strain at the higher concentrations that was similar to the level of zinc uptake at lower concentrations in the wild type strain. Since the latter were able to trigger trehalase activation as opposed to the high concentrations of zinc in the *zrt1*Δ strain, we could conclude that the signaling is not triggered by intracellular zinc but rather by Zrt1 acting as a transceptor at the level of the plasma membrane. As opposed to iron signaling by Ftr1, deletion of *ZRT1* did not completely abolish zinc-induced trehalase activation. This might indicate that another zinc transporter might also have limited transceptor functionality. A possible candidate is the low-affinity zinc transporter Zrt2, which shows strong structural and sequence similarity with Zrt1 9. In the presence of Zrt1, Zrt2 does not influence high-affinity uptake and is not expressed under zinc-limiting conditions. However, upon deletion of *ZRT1, *expression of *ZRT2 *might be enhanced under zinc-limiting conditions in order to cope with the zinc limitation and then act as a weak transceptor upon re-addition of zinc to the starved cells.

Finally, we have obtained more insight into the action mechanism of Zrt1 through mutational analysis. We found that the Zrt1^E261Q^ protein completely lacked signaling and transport capacity. Glu^261^ is a strongly conserved residue in metal ion transporters. Previous work has shown that mutagenesis of the corresponding residue, Glu^228^, in the *Arabidopsis thaliana *IRT1 metal ion transporter, another member of the ZIP family, completely abolished transport for all metal ions that are transported by this carrier 70. Hence, this residue may be essential for proper functioning of all ZIP family members. Our mutational analysis also identified a mutant allele, Zrt1^E226Q^, in which zinc uptake was partially reduced with about 70% but signaling with the same concentration of ZnCl_2_ was not affected. This partial uncoupling of transport and signaling provides further support for Zrt1 functioning as a transceptor, since it argues against the substrate in the cytosol being responsible for triggering the signaling. It is in agreement with previous results obtained with the other yeast transceptors in which transport could be abolished without affecting signaling 18,21,22. Mutagenesis of specific putative proton binding sites in Sul1, Sul2 and Pho84 abolished transport but not signaling indicating that binding of the substrate to the transporter is enough to establish the conformation that triggers signaling. Glu^226^ is also strongly conserved in ZIP family members 70, but its importance has not been addressed yet by site-directed mutagenesis. Glu^226^ is located in TMD IV, closer to the border with the cytosol than Glu^261^ in TMD V. This may allow the mutant transceptor Zrt1^E226Q^ to bind zinc and gain the conformation that triggers signaling to the PKA pathway without being able to transport the zinc ion efficiently further into the cytosol.

In conclusion, our work has identified yeast Ftr1 and Zrt1 as the first transceptors for micronutrients, iron and zinc, respectively. We have shown that deprivation for iron or zinc leads to growth arrest with development of a low-PKA phenotype and that re-addition of iron or zinc to cells starved for iron or zinc, respectively, triggers rapid activation of the well-established PKA target trehalase. The identification of Ftr1 and Zrt1 as micronutrient transceptors strengthens the concept that yeast cells use the high-affinity transporters that are generally induced upon starvation for essential nutrients as receptors upon exit from the starved stationary phase. Our finding makes it likely that all transceptors use the same or a similar signaling mechanism for activation of the PKA pathway and its elucidation may be facilitated by the current availability of a wide range of transceptors.

## MATERIALS AND METHODS

### Yeast strains

The *S. cerevisiae* strains used in this work are shown in Table 1. With exception of those used for the results in Fig. 4F, they are all isogenic with wild type strain Σ1278 (MATα *ura3*Δ). The deletion strains have been created by transferring the KanMX cassette from the strains in the Euroscarf deletion collection to the Σ1278 wild type strain. Primers used for amplification of the cassette were Ftr1 Fw (ACTACTAACTCCTGAGACA) Ftr1 Rv (ATGGTTATATCCTGGCATG), Fet3 Fw (GCTTGCCTATTTCACGGTTAC), Fet3 Rv (TCTCAGTAATCTCAGGCTATT), Zrt1 Fw (AAATGCACTAGAACATGGCG) and Zrt1 Rv (TTCATGACTATTTAAATGCCTT). The strains used for the results in Fig. 4F are isogenic with wild type strain BY4742 (MATα *his3*Δ* leu2*Δ* lys2*Δ* ura3*Δ) and with the transporter deletion strains from the Euroscarf deletion collection.

**Table 1:** *S. cerevisiae* strains used in this study.

**Strain name**	**Genotype**	**Origin/Reference**
Σ1278 (JT4500)	*MATα ura3*Δ	ResGen/Invitrogen
JS001	Σ1278 *MATα ura3*Δ* ftr1::KanMX*	This study
JS002	Σ1278 *MATα ura3*Δ* zrt1::KanMX*	This study
JS003	Σ1278 *MATα ura3*Δ* ctr1::KanMX*	This study
JS004	Σ1278 *MATα ura3*Δ* fet3::KanMX*	This study
JS005	Σ1278 *MATα ura3*Δ* Ftr1-GFP-KanMX*	This study
JS006	Σ1278 *MATα ura3*Δ* Zrt1-GFP-KanMX*	This study
JS007	Σ1278 *MATα ura3*Δ* Ctr1-GFP-KanMX*	This study
JS008	Σ1278 *MATα ura3*Δ* Ftr1-HA-KanMX*	This study
JS009	Σ1278 *MATα ura3*Δ* Zrt1-HA-KanMX*	This study
JS010	Σ1278 *MATα ura3*Δ* Ctr1-HA-KanMX*	This study
JS011	Σ1278 *MATα*	This study
BY4742	*MATα his3*Δ* leu2*Δ* lys2*Δ* ura3*Δ	Euroscarf
	*BY4742 ftr1*Δ	Euroscarf
	*BY4742 smf2*Δ	Euroscarf
	*BY4742 mrs3*Δ	Euroscarf
	*BY4742 smf1*Δ	Euroscarf
	*BY4742 fet4*Δ	Euroscarf
	*BY4742 fet3*Δ	Euroscarf

### Growth media and starvation conditions

To obtain iron- or zinc-starved cells, the cells were first cultured at 30(C into exponential phase (OD_600nm _( 1.5 - 2) in all trace medium with 2% (w/v) glucose. The composition of all trace medium is based on CSM complete medium and contains the list of chemicals shown in Table 2. The medium was prepared by mixing all components except vitamins together in milliQ water and then autoclaved. A 500x filter-sterilized vitamin stock was prepared separately and the appropriate amount added to the medium right before use. Exponential-phase cells were harvested and suspended in starvation medium supplemented with a specific chelator and containing 4% glucose. They were incubated under shaking for 24, 48 or 72 h at 30(C. Care was taken that the glucose level remained high (>2%) throughout the incubation. For iron starvation medium, ferric chloride was omitted from the all trace medium and the medium was supplemented with 80 μM BPS or 500 μM ferrozine. For zinc starvation medium, zinc sulfate was excluded from the all trace medium and the medium was supplemented with 1 mM EDTA and 10 mM citrate.

**Table 2:** Composition of all trace medium.

**Component**	**Concentration**	**Component**
Ammonium sulfate	5 g/L	Ammonium sulfate
Biotin	0.002 mg/L	Biotin
Calcium pantothenate	0.4 mg/L	Calcium pantothenate
Folic acid	0.002 mg/L	Folic acid
Inositol	2 mg/L	Inositol
Niacin	0.4 mg/L	Niacin
P-aminobenzoic acid	0.2 mg/L	P-aminobenzoic acid
Pyridoxine hydrochloride	0.4 mg/L	Pyridoxine hydrochloride
Riboflavin	0.2 mg/L	Riboflavin
Thiamine hydrochloride	0.4 mg/L	Thiamine hydrochloride
Boric acid	0.5 mg/L	Boric acid
Copper sulfate	0.04 mg/L	Copper sulfate
Potassium iodide	0.1 mg/L	Potassium iodide
Ferric chloride (omitted in –Fe)	0.2 mg/L	Ferric chloride (omitted in –Fe)
Manganese sulfate	0.4 mg/L	Manganese sulfate
Sodium molybdate	0.2 mg/L	Sodium molybdate
Zinc sulfate (omitted in –Zn)	0.4 mg/L	Zinc sulfate (omitted in –Zn)
Magnesium sulfate	1 g/L	Magnesium sulfate
Sodium chloride	100 mg/L	Sodium chloride
Potassium phosphate dihydrate	1.75 g/L	Potassium phosphate dihydrate
Di-potassium hydrogen phosphate trihydrate	0.25 g/L	Di-potassium hydrogen phosphate trihydrate
Calcium chloride	120 mg/L	Calcium chloride
Uracil	50 mg/L	Uracil
Glucose	2% (w/v)	Glucose

### Growth curves and budding percentage

100 ml cultures were grown under continuous shaking at 30°C, in the indicated medium. At regular time intervals, a 1 ml sample was taken for OD_600nm _measurement with a Biophotometer from Eppendorf.

### RNA extraction and qPCR

For determination of *FTR1* and *ZRT1 *expression, appropriately-starved cells were collected, spun down, and the pellets were frozen in liquid nitrogen and stored at -80°C. Total RNA was isolated by phenol extraction and treated with RNAse free DNAse (Roche). cDNA was prepared following the instructions of the Reverse Transcription Kit by Promega A3500. Subsequently, relative quantification of *FTR1/ZRT1* expression and the reference genes *18S* and *ACT1 *was performed using real-time PCR with a GoTaq qPCR Master Mix from Promega for a StepOnePlus System (Applied Biosystems). The primers used were: Fw Ftr11 (CCTTGTCTGTGGCGACGTT), Rv Ftr1 (CTACGAACTTCCCGAGCAAACTA), Fw Zrt1 (GCCATCGGTTTGGGTGTTC), Rv Zrt1 (CCAGAGATAACAAGCGCAGTGT).

### Fluorescence microscopy

The fluorescence-based localization studies of Ftr1-GFP and Zrt1-GFP were performed with iron or zinc-starved cells, respectively, and carried out using an Olympus FV1000 confocal laser scanning microscope. Images were processed with the accompanying software, FV10-ASW 2.0.

### Biochemical determinations

Trehalose and glycogen content were determined using previously described protocols, in which trehalose and glycogen are converted to glucose by trehalase and β-amyloglucosidase, respectively and subsequently determined using the GOD-PAP method 17. Trehalase activity after addition of iron or zinc was determined as previously described 17. The specific activity of trehalase is expressed as nmol glucose liberated*min^-1^*(mg protein)^-1^. Total amount of protein in the samples was determined using the standard Lowry method.

### Determination of iron and zinc uptake

Cells were starved either for iron using the BPS chelator, or for zinc using the EDTA chelator as described above. Subsequently, cells were washed once with 25 mM MES buffer pH 6.0 and resuspended in starvation medium (without chelators) to a density of 60 mg/mL. After acquiring blank samples, 40 μL of cells were incubated for each sample in 3-fold for 10 min at 30°C. Subsequently, 10 μL of a 5x concentrated stock of the indicated concentration of FeCl_3_ (containing a specific activity of 3,000 cpm/nmol Fe^55^) or ZnCl_2_ (containing a specific activity of 3,000 cpm/nmol Zn^65^) was added to the samples. The samples were incubated for 1 min in the presence of the nutrient after which uptake was stopped by addition of 10 mL ice-cold unlabelled FeCl_3_ or ZnCl_2_ equal to the highest concentration used in the experiment. The sample was filtered over a glass microfiber filter (Whatman GF/C) prewet with the unlabelled FeCl_3_ or ZnCl_2_. The filter was immersed in scintillation fluid in a scintillation tube and the Fe^55^ or Zn^65^ content was determined using a Hidex 300 SL Liquid Scintillation Counter. Uptake was expressed as nmol*min^-1^*(mg dry weight)^-1^.

### Site-directed mutagenesis

To obtain the point mutant forms of Zrt1, site-directed mutagenesis was used. Full-length *ZRT1* was cloned into the YCplac33 plasmid using the SalI (Fw) and XmaI (Rev) restriction sites using the primers Zrt1-Sal1 Fw TTTTTTTGTCGACAGACTTGAGATAGATGTACC and Zrt1-XmaI Rv TTTTTTCCCGGGAGTGGTCAATGAGATCAAA. Subsequently, site-directed mutagenesis was performed using primers containing the specific mutations as indicated in the primer list in Table 3, in combination with the Q5 High Fidelity DNA Polymerase and a PCR reaction containing 18 cycles adjusted to the conditions used. This PCR amplification yielded a plasmid construct carrying *ZRT1* with the desired mutation. The original plasmid was subsequently digested with 1 μL of the DpnI restriction enzyme, leaving only the newly synthesized plasmids in intact form. The newly constructed plasmids were subsequently transformed into competent *E. coli* cells, the transformants selected based on ampicillin resistance, the plasmids purified and finally transformed into the Σzrt1Δ strain.

**Table 3:** List of primers used for site-directed mutagenesis of Zrt1.

**Name**	**Mutation**	**Sequence**
Zrt1-D105N Fw	ZRT1^D105N^	CACTTAATGAACCCTGCTTAT
Zrt1-D105N Rv	ZRT1^D105N^	ATAAGCAGGGTTCATTAAGTG
Zrt1-D144N Fw	ZRT1^D144N^	TTCCTTACTAATCTATTCAGT
Zrt1-D144N Rv	ZRT1^D144N^	ACTGAATAGATTAGTAAGGAA
Zrt1-E226Q Fw	ZRT1^E226Q^	TTAATTTTACAATTCGGTGTG
Zrt1-E226Q Rv	ZRT1^E226Q^	CACACCGAATTGTAAAATTAA
Zrt1-E261Q Fw	ZRT1^E261Q^	CAATCATTTCAAGGTTTAGGT
Zrt1-E261Q Rv	ZRT1^E261Q^	ACCTAAACCTTGAAATGATTG
Zrt1-E273Q Fw	ZRT1^E273Q^	TCAGCCATTCAATTCCCTAGA
Zrt1-E273Q Rv	ZRT1^E273Q^	TCTAGGGAATTGAATGGCTGA
Zrt1-D322N Fw	ZRT1^D322N^	GGTGTTTTGAATGCCATTTCT
Zrt1-D322N Rv	ZRT1^D322N^	AGAAATGGCATTCAAAACACC
Zrt1-E336Q Fw	ZRT1^E336Q^	GGTTTGGTTCAACTACTAGCA
Zrt1-E336Q Rv	ZRT1^E336Q^	TGCTAGTAGTTGAACCAAACC
Zrt1-E354Q Fw	ZRT1^E354Q^	GATCTAAGACAATTGTCCTTC
Zrt1-E354Q Rv	ZRT1^E354Q^	GAAGGACAATTGTCTTAGATC

### Reproducibility of the results

All experiments were repeated at least two times. Standard deviations are shown for comparisons between independent data points (transport measurements). Time-course experiments of metal-ion induced trehalase activation were repeated three to five times. Representative results are shown for these comparisons between collections of interdependent data points.
